# Supplementary material for: Quasi-two-dimensional superconductivity from dimerization of atomically ordered AuTe2Se4/3 cubes
Source: Nat Commun. 2017 Oct 11;8:871. doi: 10.1038/s41467-017-00947-0 (PMC5636790; doi:10.1038/s41467-017-00947-0)
Supplement: Supplementary file 1 — Supplementary Information [file 41467_2017_947_MOESM1_ESM.pdf]

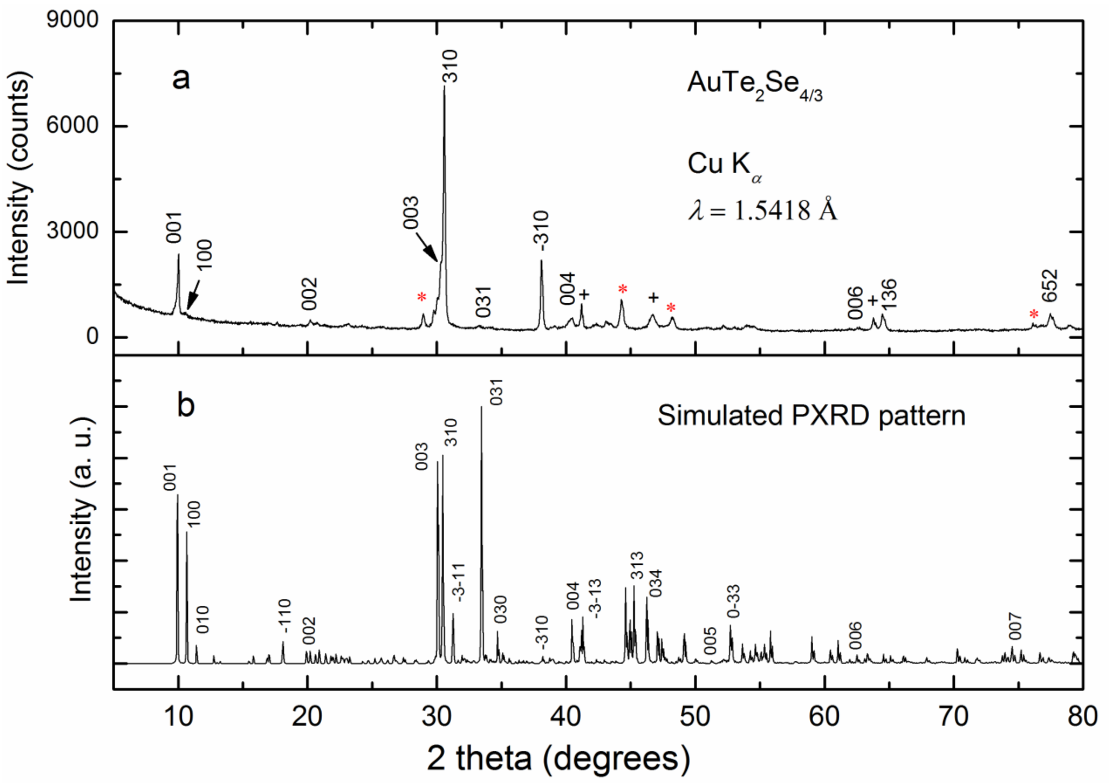

Supplementary Figure 1. The experimental and simulated PXRD patterns for $AuTe_2Se_{4/3}$. (a): The experimental powder X-ray diffraction (PXRD) pattern of $AuTe_2Se_{4/3}$ taken at room temperature. (b) The simulated PXRD pattern based on the determined crystal structure of $AuTe_2Se_{4/3}$. It can be seen that the main peaks of the experimental pattern can match the simulated peaks, except minor peaks from impurity labeled as red stars (*). The broad peaks, labeled as (+), cannot accurately be indexed due to multiple possible peaks around this angle. Besides, there are high intensities for (001) and (310) peaks and the usually low intensities for (100) and (031) peaks in the experimental pattern, which cannot be used to determine crystal structure.

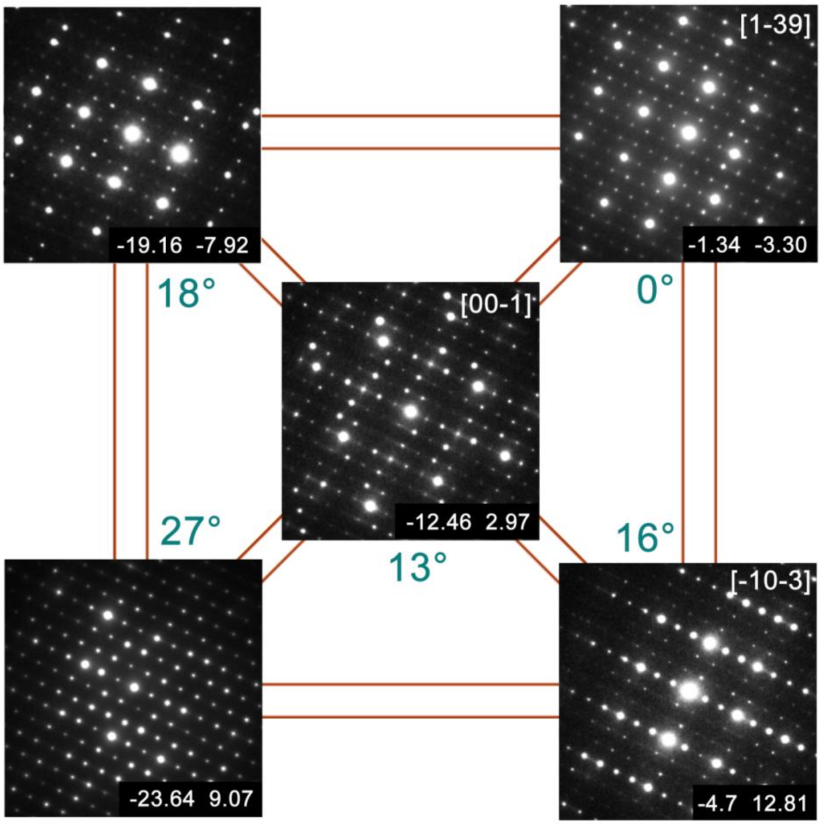

Supplementary Figure 2. All the SAED images from different angle long Kikuchi lines. The SAED images from different tilt angle along three Kikuchi lines. The angle values are directly read from the parameter of STEM. We use the combinations of [00-1], [1-39] and [-10-3] SAED patterns and corresponding HAADF images to determine the whole crystal structure.

Supplementary Table 1. Crystallographic parameters of $AuTe_2Se_{4/3}$ at room temperature directly determined from TEM images.

Formula	$AuTe_2Se_{4/3}$
Temperature (K)	297
Space group	*P-1*
a (Å)	8.8457
b (Å)	8.4300
c (Å^3)	9.2793
α	77.24º
β	95.53º
γ	72.36º
Z	2
Atomic parameters:	
Au1	0.44449, 0.58552, 0.20258
Au2	0.59589, 0.16890, 0.50419
Au3	0.20051, 0.49993, 0.50074
Te1	0.29640, 0.16884, 0.50493
Te2	0.14501, 0.58545, 0.20333
Te3	0.54038, 0.25442, 0.20678
Te4	0.89538, 0.16897, 0.50345
Te5	0.74398, 0.58558, 0.20184
Te6	0.34860, 0.91661, 0.19839
Se1	0.24090, 0.25436, 0.20752
Se2	0.83987, 0.25449, 0.20604
Se3	0.04912, 0.91655, 0.19913
Se4	0.64809, 0.91668, 0.19765

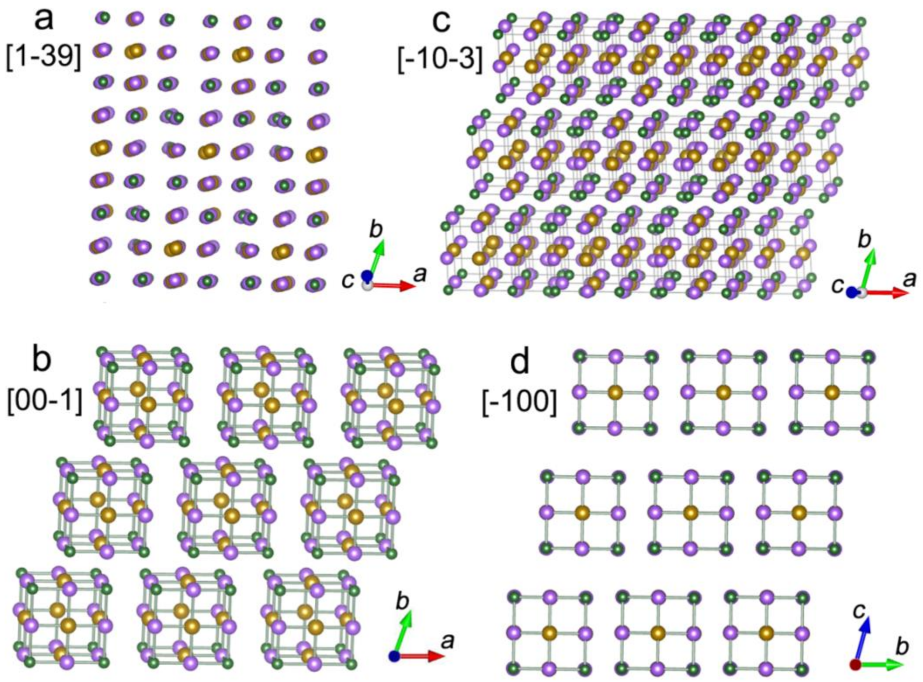

Supplementary Figure 2. Resolved crystal structure viewed from different zone axes. The schematic crystal structure viewed from (a) [1-39], (b) [00-1], (c) [-10-3] and (d) [-100] directions, respectively. It can be seen that these atomic distributions perfectly match the HAADF images of STEM measurement.

Supplementary Note 1.

Analyses of the superconducting fluctuations

The analyses of ρ(T) curve were performed based on the model of superconducting amplitude fluctuation, using the expression,

$$\rho(T) = (\frac{1}{\rho_{\mathrm{N}}} + \Delta G_{\mathrm{SF}}), \qquad (1)$$

$$\frac{1}{\rho_{\mathrm{N}}} = \frac{1}{a+bT^2} + cln\left(\frac{T}{T_0}\right), \qquad (2)$$

$$\Delta G_{\mathrm{SF}} = \Delta G_{\mathrm{AL}} + \Delta G_{\mathrm{MT}} + \Delta G_{\mathrm{DOS}} = \frac{e^2}{16\hbar}\left(\frac{T_{\mathrm{c0}}}{T-T_{\mathrm{c0}}}\right) + \frac{e^2}{8\hbar}\frac{T_{\mathrm{c0}}}{\{T(1-\delta)-T_{\mathrm{c0}}\}}\ln\left(\frac{T-T_{\mathrm{c0}}}{\delta T}\right), \qquad (3)$$

Where a, b, c and T_0 are T-independent fitting parameters. The term of ΔG_{SF} in Supplementary Eq. 1 contains three principle contributions [1]: the Aslamazov-Larkin (AL) process corresponding to the direct effect of thermal fluctuation [2] and the anomalous Maki-Thompson (MT) process corresponding to the indirect effect of transition from Copper pairs into quasiparticles [3, 4]. The second term of Supplementary Eq. 2 represents the effect of weak localization (WL) and the Coulomb interaction between particles with nearly identical momenta (ID) [5]. The term of ΔG_{DOS} describes the depression of density of states (DOS) induced by thermal fluctuation [6, 7]. In the Supplementary Eq. 3, the fitted T_{c0} is the threshold temperature at which the finite amplitude of the order parameters develops, and δ is the pairing-breaking parameter. The experimental data are fitted by equation Supplementary Eq. 1, and the obtained parameters are shown in Supplementary Fig. 4.

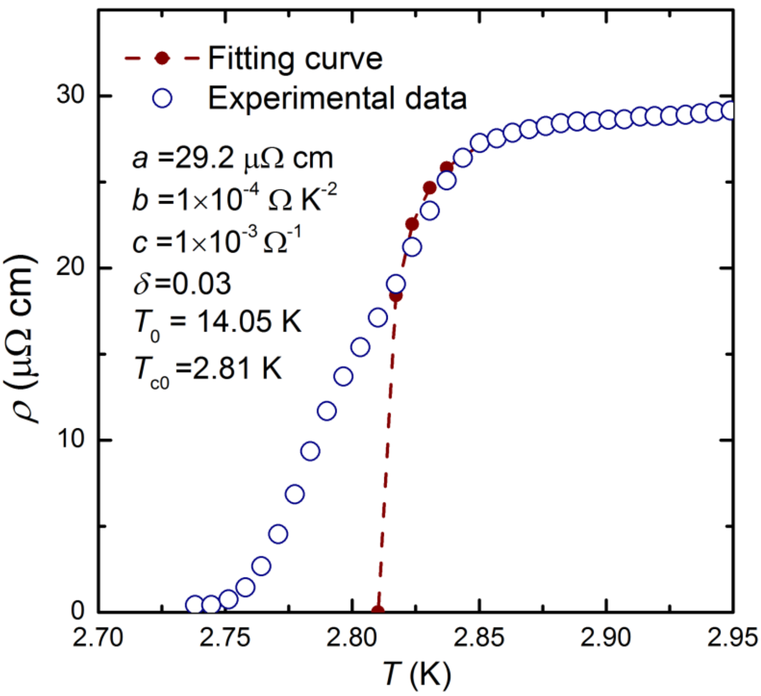

Supplementary Figure 4. Superconducting fluctuations around T_c. Temperature dependent electrical resistivity measured without magnetic field in the superconducting transition range. The open cycle symbol and the dashed line denote the experimental data and fitting curve, respectively.

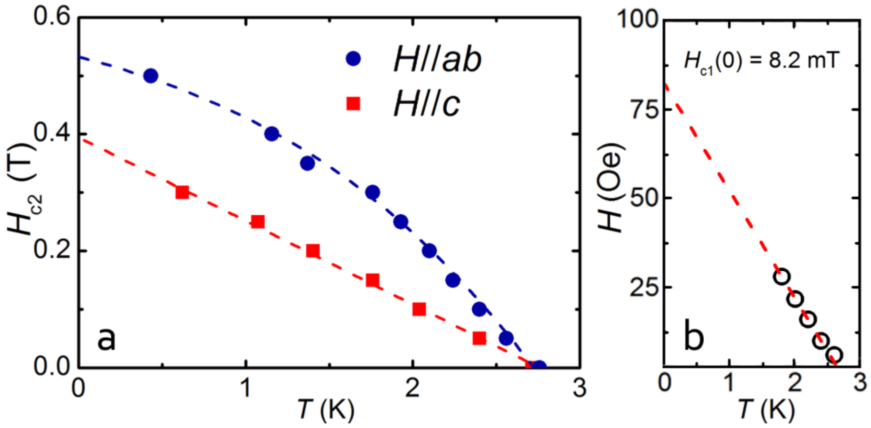

Supplementary Figure 5. Upper and lower critical fields. (a) Two upper critical fields along ab and c direction are estimated by the 2D Ginzburg- Landau equations as discussed in the main text. It can be seen that the sample shows an anisotropic as magnetic field along different direction. (b) The lower critical fields estimated from linear fitting equation. It can be found that this value, 8.2mT, is smaller than the upper critical fields, indicating a type-II superconductor.

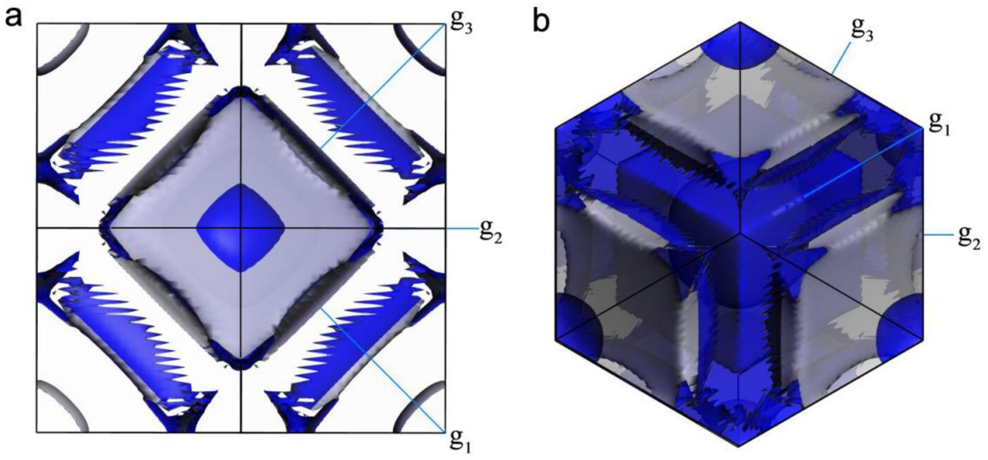

Supplementary Figure 6. The Fermi surface of metastable $Au_{1-x}Te_x$ phase. The 3D Fermi surface of disorder $Au_{1-x}Te_x$ from (a) top and (b) stereo view. The central Fermi surface shows 3D type and the outer Fermi sheets exhibit dented plane feature, which is totally different from that of ordered $AuTe_2Se_{4/3}$.

Supplementary References:

1. Glatz, A., Varlamov, A. A., Vinokur, V. M. Fluctuation spectroscopy of disordered two dimensional superconductors. *Phys. Rev. B* **84**, 104510 (2011).
2. Aslamazov, L. G., Larkin, A. I. The influence of fluctuation pairing of electrons on the conductivity of normal metal. *Phys. Lett.* **26A**, 238–239 (1968).
3. Maki, K. The critical fluctuation of the order parameter in type-II superconductors. *Prog. Theor. Phys.* **39**, 897–906 (1968).
4. Thompson, R. S. Microwave, flux flow, and fluctuation resistance of dirty type-II superconductors. *Phys. Rev. B* **1**, 327–333 (1970).
5. Baturina, T. I., Postolova, S. V., Mironov, A. Y., Glatz, A., Baklanov, M. R., Vinokur, V. M., Superconducting phase transitions in ultrathin TiN films. Europhys. Lett 97, 17012 (2012).
6. Ioffe, L. B., Larkin, A. I., Varlamov, A. A., Yu, L. Effect of superconducting fluctuations on the transverse resistance of high-*T*c superconductors. *Phys. Rev. B* **47**, 8936–8941 (1993).
7. Dorin, V. V., Klemm, R. A., Varlamov, A. A., Buzdin, A. I., Livanov, D. V. Fluctuation conductivity of layered superconductors in a perpendicular magnetic field. *Phys. Rev. B* **48**, 12951–12965 (1993).
